# Effectiveness and economic impact of Dupilumab in asthma: a population-based cohort study

**DOI:** 10.1186/s12931-023-02372-y

**Published:** 2023-03-07

**Authors:** Paola Faverio, Raffaella Ronco, Matteo Monzio Compagnoni, Matteo Franchi, Giovanni Franco, Giulia Bonaiti, Martina Bonifazi, Federico Mei, Fabrizio Luppi, Alberto Pesci, Giovanni Corrao

**Affiliations:** 1 Department of Medicine and Surgery, School of Medicine and Surgery, Università Degli Studi di Milano Bicocca, Monza, Italy; 2 National Centre for Healthcare Research and Pharmacoepidemiology, University of Milano-Bicocca, Milan, Italy; 3 Laboratory of Healthcare Research and Pharmacoepidemiology, Unit of Biostatistics, Epidemiology and Public Health, Department of Statistics and Quantitative Methods, University of Milano-Bicocca, Milan, Italy; 4 Department of Biomedical Sciences and Public Health, Universitá Politecnica Delle Marche-Respiratory Diseases Unit, Azienda Ospedaliero-Universitaria Ospedali Riuniti, Ancona, Italy; 5 Respiratory Unit, Fondazione IRCCS San Gerardo Dei Tintori, Via Pergolesi 33, 20900 Monza, Italy

**Keywords:** Asthma, Clinical allergy and immunology, Inflammation

## Abstract

**Rationale:**

Severe asthma is burdened by relevant socio-economic and clinical impact. Randomized controlled trials on Dupilumab showed efficacy and a good safety profile, but post-market studies are needed.

**Objectives:**

To evaluate the impact of Dupilumab on (i) the use of anti-asthmatic drugs, including oral corticosteroids (OCS), (ii) the rates of asthma exacerbation-related hospital admissions, and (iii) the healthcare costs in patients with asthma.

**Methods:**

Data were retrieved from Healthcare Utilization database of Lombardy region (Italy). We compared healthcare resources use between the 6 months after Dupilumab initiation (“post-intervention period”) and (i) the 6 months before Dupilumab initiation (“wash-out period”) and (ii) the corresponding 6 months of the prior year (“pre-intervention period”).

**Main results:**

In a cohort of 176 patients, Dupilumab significantly reduced anti-asthmatic drugs use (including OCS and short-acting β2-agonists, inhaled corticosteroids (ICS)/long-acting β2-agonists and ICS alone) when comparing the “pre-intervention” to the “post-intervention” period. When considering hospital admissions, we observed a not statistically or marginally significant reduction between both periods before Dupilumab and the post-intervention period. Six-months discontinuation rate was 8%. Overall healthcare costs had a tenfold increase between the “pre-intervention” and “post-intervention” period, which was mainly led by the biologic drug cost. Conversely, expenditures connected to hospital admissions did not change.

**Conclusions:**

Our real-world investigation suggests that Dupilumab reduced anti-asthmatic drugs use, including OCS, in comparison to a corresponding period in the prior year. However, long-term healthcare sustainability remains an open issue.

**Supplementary Information:**

The online version contains supplementary material available at 10.1186/s12931-023-02372-y.

## Introduction

Asthma is a chronic respiratory disease with a prevalence ranging between 1 and 18% in high-income countries [1]. About 5 to 10% of patients with asthma show severe asthma, defined as a form of asthma that is uncontrolled despite treatment with high-dose inhaled corticosteroids (ICS) and long-acting β2-agonists (LABA) [2].

The costs associated with asthma management increase as disease control worsens. In fact, about half of asthma expenditure in Italy is attributable to 25% of patients with uncontrolled asthma and similar data are reported in other European countries [3].

In the last decade, treatment for severe asthma has been largely improved by the availability of new targeted therapies, modulating specific cell signaling pathways. Particularly, Dupilumab, a fully human anti–interleukin-4 receptor α monoclonal antibody that blocks both interleukin-4 and interleukin-13 signaling, has been recently licensed as add-on therapy for severe asthma, and is also approved for the treatment of atopic dermatitis and nasal polyposis.

Randomized controlled trials (RCT) and the first observational studies available suggest that dupilumab greatly reduces oral corticosteroids (OCS) use and exacerbation-related hospitalizations, [4–6] while tolerability, patients’ selection and the economic impact on the healthcare service are still a matter of debate.

Furthermore, given the recent introduction in clinical practice of Dupilumab for severe asthma and nasal polyposis (December 2020 in Italy), real-world cost-effectiveness studies are still lacking.

The aim of this study was to evaluate the impact of Dupilumab on (i) the use of anti-asthmatic drugs other than biologics, including OCS, (ii) the rates of asthma exacerbation-related hospital admissions, both hospitalizations and emergency room (ER) visits, and (iii) the healthcare costs, on a large population-based cohort of patients with asthma. Discontinuation of Dupilumab at 6 months after treatment initiation was also evaluated.

## Materials and methods

### Setting and cohort selection

This study was based on computerised Healthcare Utilization (HCU) databases of Lombardy, an Italian northern region accounting for almost 10 million people (about 16% of the national whole population). In Lombardy, the management of the National Health Service (NHS) has been associated since 1997 with an automated system of HCU databases which includes a variety of information on the beneficiaries of the regional health service (virtually all residents in the region), such as (i) demographic data, (ii) drug prescriptions dispensed outside or directly administered in hospital(s), (iii) specialistic visits, diagnostic exams provided by the NHS, and ER admissions [7]. Details of HCU databases in the field of respiratory diseases have been reported in more details elsewhere [8]. Diagnostic procedures and drugs codes used in the current study for drawing records and fields from the considered databases are reported in Additional file 2: Table S1.

**Fig. 1:**
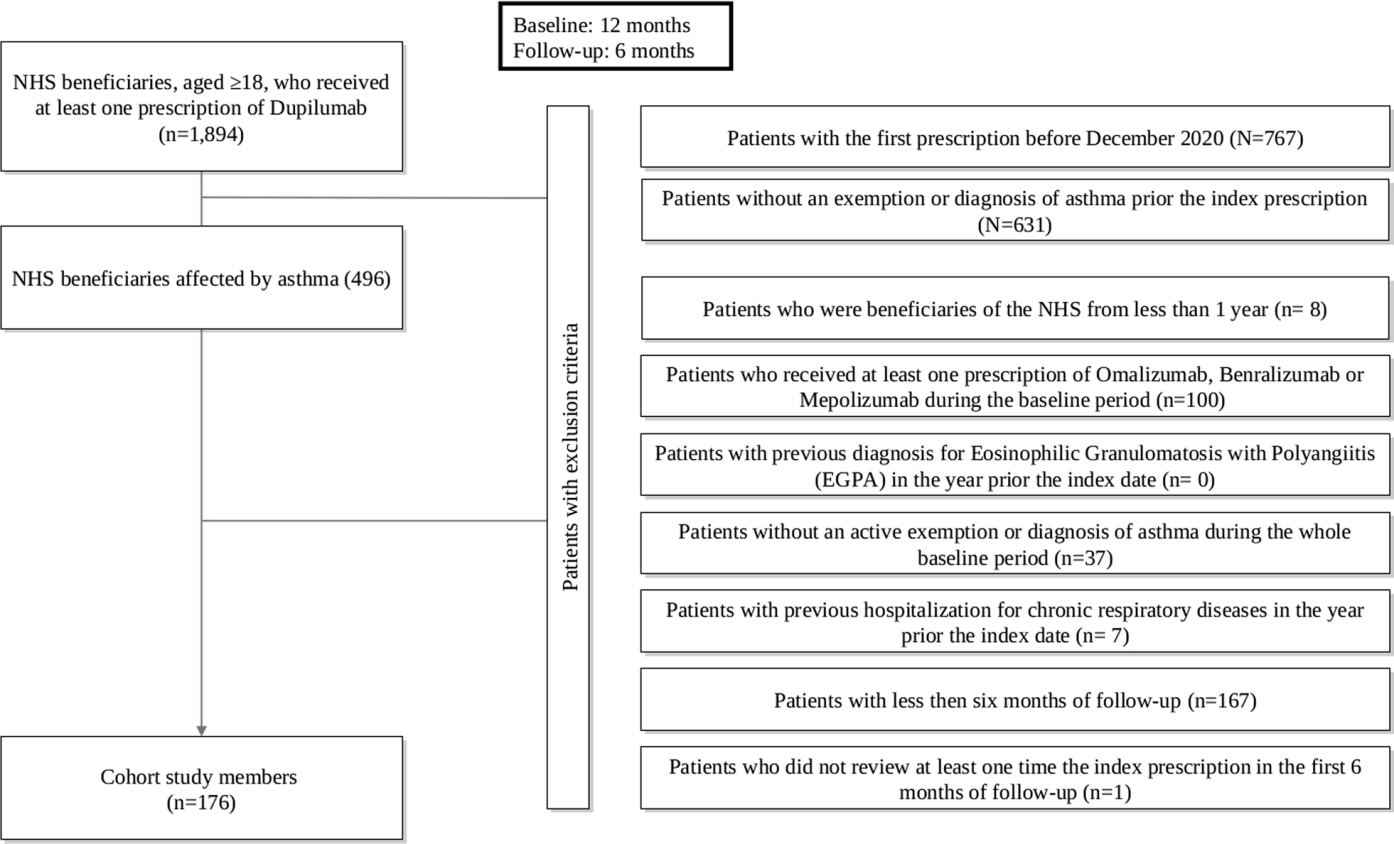
Flow-chart of inclusion and exclusion criteria for the eligibility of patients with a diagnosis of asthma and receiving at least one prescription of Dupilumab during the period December, 1st 2020 and July, 31st 2021. ^¥^Patients (i) with an active exemption, or (ii) who experienced a previous hospital admission with diagnosis for asthma or asthma-related respiratory problems

The target population consisted of all the residents beneficiaries of the regional health service (RHS) aged 18 or older. Those who, between December, 1st 2020 and July, 31st 2021, received at least one prescription of Dupilumab were identified. The date of their first Dupilumab administration during the recruitment period was recorded as the index date. The selection criteria for the study cohort are summarized in Fig. 1. In particular, patients who experienced hospital admissions in the year prior the index date for chronic respiratory diseases other than asthma were excluded, in order to minimize possible confounders. The list of chronic respiratory diseases considered in the cohort selection is reported in Additional file 2: Table S1.

### Study design and outcomes

**Fig. 2:**
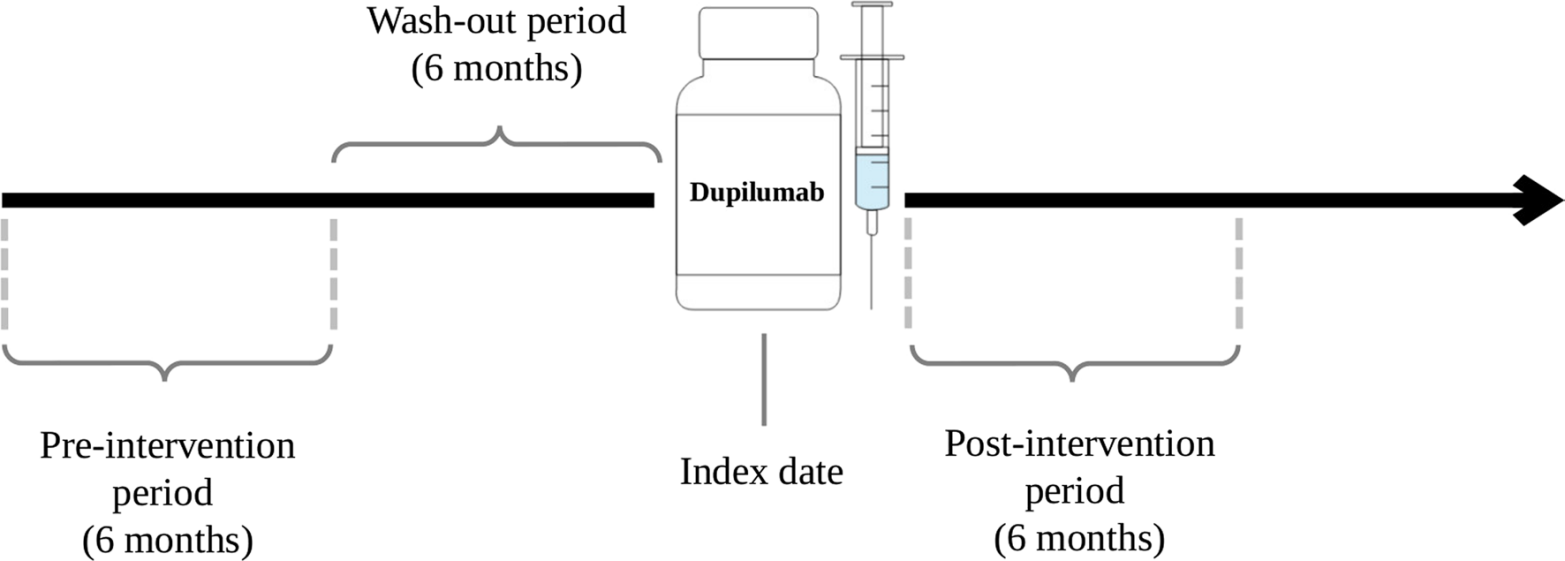
Study design. Index date: date of the first prescription of Dupilumab

For each cohort member, starting from the index date, 3 periods of observation were considered, as shown in Fig. 2. The first one, so-called the “pre-intervention period”, was defined as the semester starting exactly one year before the index date, the second one, “the wash-out period”, was defined as the semester immediately preceding the index date, while the third and last one, so-called the “post-intervention period”, was defined as the 6 months immediately following the index date. In all three periods, all medications, outpatient visits, and hospital admissions were recorded.

The exposure was the treatment with Dupilumab, whereas the outcome of interest was the use of anti-asthmatic medications, other than biologic drugs, during the post-intervention period compared to the use during the pre-intervention and wash-out periods. The use of anti-asthmatic drugs was assessed with several measures. The two main primary outcomes were the change comparing the post-intervention period to the pre-intervention period in (i) the percentage of patients with at least one anti-asthmatic drug prescription (*“any anti-asthmatic use*”), and (ii) the mean number of anti-asthmatic drug prescriptions per patient. These changes were also measured for OCS prescriptions *(“any OCS use”*) and hospital admissions (including ER visits) for asthma exacerbations, as secondary outcome of the study. Moreover, the post-intervention period was also compared with the wash-out period.

Furthermore, as one of the secondary outcomes, we evaluated treatment discontinuation of Dupilumab at 6 months after treatment initiation. Dupilumab prescriptions were considered ‘consecutive’ if the interval between the end of one prescription and the start of the following one was less than 61 days, and ‘interrupted’ otherwise; interrupted prescriptions were considered to lead to discontinuation of treatment.

Concerning the other secondary aims of our study, healthcare costs were assessed from the amount that the Regional Health Authority reimbursed to health providers for healthcare services and available in our databases. Costs included hospital admissions (considering also ER visits), drugs dispensed by the NHS and outpatient services, and were assessed separately for all the respiratory related and non-respiratory related healthcare services provided by the RHS, respectively. With the aim of expressing cost as a rate, healthcare costs accumulated overall by the cohort were divided by the number of person-years accumulated from that cohort during the cost-related periods. The average 6-month healthcare cost was calculated and expressed in Euros every person-year. The change of this measure between the pre-intervention and the post-intervention period was estimated.

Finally, because the intensity of healthcare may vary along calendar time for the seasonality of medical services, a sensitivity analysis to account for the seasonality effect and confirm the robustness of our findings was performed. A reference cohort suitable to be used as a comparator for the study cohort was generated. Patients who were eligible to be selected as comparators were those who had a diagnosis of asthma and were not in treatment with Dupilumab during the recruitment period. For each study cohort member, one eligible comparator was randomly selected to be matched for gender, age at index date and date of asthma diagnosis (± 180 days). The main analyses were replicated on the cohort of comparators and the variations on outcome measures between the pre-intervention or wash-out periods and the post-intervention period were compared with those obtained in the study cohort.

### Data analysis

Continuous variables were described as mean and standard deviation (SD), whereas absolute frequencies and percentages were reported for categorical variables. Comparisons of outcome measures between pre-intervention and post-intervention period were performed using (i) the χ2 test for categorical variables, and (ii) the Student’s *t-test* for the means of paired samples for continuous variables. The software SAS (version 9.4 for Windows; SAS Institute, North Carolina, USA) was used to perform all analyses. For all hypotheses tested, two-tailed p-values less than 0.05 were considered significant.

The “Materials and methods” section of this manuscript partially overlaps with another study published by our group on the economic impact of anti-IL-5 agents in severe asthma [9].

## Results

Out of the 496 patients affected by asthma who received at least one prescription of Dupilumab, 100 (20.2%) were excluded because they had received at least one prescription of Omalizumab, Benralizumab or Mepolizumab during the pre-intervention period, Fig. 1. In particular, 37 patients received Benralizumab, 36 Mepolizumab, 4 Omalizumab, and 3 subjects received a consecutive prescription of two medications (omalizumab + mepolizumab in one case, omalizumab + benralizumab in one case, and mepolizumab + benralizumab in the last case).

One hundred seventy-six patients (48.3% women, mean (SD) age 47.1 (15.5) years) with asthma receiving at least one prescription of Dupilumab met all the inclusion criteria and were included in the final analysis, Fig. 1. The majority of patients (150, 79.6%) had a diagnosis of asthma ≥ 5 years earlier, presented a low burden of comorbidities assessed by the Multisource Comorbidity Score (MCS) (an index of patients’ clinical status, provided by the regional Italian data and validated for outcome prediction [7]), with MCS ≤ 5, meaning good or optimal clinical profile, in 171 (97.2%) patients. Main co-medications prescribed included antihypertensives (45–25.6% of cases), antithrombotics (13–7.4%) and antidepressants (10–5.7%).

In regard to study outcomes, the overall number of patients receiving at least one prescription of anti-asthmatic drugs other than biologic agents over a 6-month period decreased by 23.6% comparing the 6 months after Dupilumab initiation “post-intervention period” to the corresponding 6 months of the previous year “pre-intervention period” (68.6% *vs* 89.7%, respectively, p < 0.001), and the mean number of prescriptions per patients decreased by 50.9%, as shown in Table 1. The number of patients with at least one prescription for each pharmacological class of anti-asthmatic drugs other than OCS is summarized in Table 2. The number of patients with at least one claim of short-acting β2-agonists (SABA), considered as reliever medications for asthma flare-ups, significantly decreased after Dupilumab initiation (33.5% *vs* 12.5%, p-value < 0.001). The number of patients with at least one claim of leukotriene receptor antagonists, considered a second-line controller option at various steps of disease severity or an add-on therapy in case of severe asthma, also significantly decreased after Dupilumab initiation (26.7% in the pre-intervention period *vs* 15.3% in the post-intervention). Finally, we found a significant decrease in the number of patients with at least one claim of ICS alone and ICS/LABA association in the pre-intervention period compared to the post-intervention (18.8% *vs* 5.1% for ICS and 80.1% *vs* 58.5% for ICS/LABA, respectively). However, when comparing the use of the same anti-asthmatic drugs between the 6 months immediately before and after Dupilumab prescription (wash-out *vs* post-intervention period) no differences were observed, Table 2.

**Table 1:** Use of specific healthcare services during the 6 months after the start of Dupilumab treatment (post-intervention period), during the corresponding 6 months of the prior year (pre-intervention period) and during the 6 months preceding the start of Dupilumab (wash-out period)

	Pre-intervention period	Post-intervention period	Absolute (%) reduction	p-value^†^
Patients with at least one asthma prescription	157 (89.7%)	120 (68.6%)	37 (23.6%)	< 0.001
Mean number of asthma prescriptions per patient (*patients with at least one Rx*)	11.3	5.5	5.7 (50.9%)	< 0.001
Patients with at least one systemic glucorticoid prescription	81 (46.0%)	29 (16.5%)	52 (64.2%)	< 0.001
Mean number of glucorticoid prescriptions per patient (*patients with at least one Rx*)	4.8	4.4	0.4 (8.56%)	0.17
Patients with at least one hospital admissions^**§**^ for asthma exacerbations	9 (5.1%)	5 (2.9%)	4.0 (44.4%)	0.28
Mean number of hospital admissions^**§**^ for asthma exacerbations^**¥**^	1.8	1.8	0.0 (0.0%)	0.73

	Wash-out period	Post-intervention period	Absolute (%) reduction	p-value^†^
Patients with at least one asthma prescription	130 (73.9%)	120 (68.6%)	10 (7.0%)	0.24
Mean number of asthma prescriptions per patient (*patients with at least one Rx*)	4.7	5.5	− 0.8 (− 17.0%)	0.12
Patients with at least one systemic glucorticoid prescription	56 (31.8%)	29 (16.5%)	27 (48.1%)	0.001
Mean number of glucorticoid prescriptions per patient (*patients with at least one Rx*)	3.0	4.4	− 1.4 (− 46.0%)	0.18
Patients with at least one hospital admissions^§^ for asthma exacerbations	14 (8.0%)	5 (2.9%)	9 (64.3%)	0.04
Mean number of hospital admissions^§^ for asthma exacerbations^¥^	1.7	1.8	− 0.1 (− 5.9%)	0.92

^†^P-value for the comparisons of outcome measures between pre-intervention and post-intervention period (i.e., p-value of χ^2^ test for categorical variable or of the Student’s t-test for the means of paired samples for continuous variables). ^§^Hospital admissions, also including ER accesses, for asthma exacerbations. ^¥^On patients who experienced at least one hospital admission, or ER access, for asthma exacerbations

**Table 2:** Use of specific anti-asthmatic drugs (expressed as the number of distinct patients treated with at least one prescription) during the 6 months after the start of Dupilumab (post-intervention period), during the corresponding 6 months of the prior year (pre-intervention period) and during the 6 months preceding the start of Dupilumab (wash-out period)

Specific anti-asthmatic drug therapy	Pre-intervention period	Post-intervention period	Absolute (%) reduction	p-value^†^
Beta-2 agonists				
Short acting	59 (33.5%)	22 (12.5%)	37 (62.7%)	< 0.001
Long acting	1 (0.6%)	1 (0.6%)		1.00
Extra-long acting	0 (0%)	0 (0%)		–
Beta-2 agonists + inhaled Corticosteroids	141 (80.1%)	103 (58.5%)	38 (27.0%)	< 0.001
Beta-2 agonists + antimuscarinic agents	4 (2.3%)	2 (1.1%)	2 (50.0%)	0.410
Beta-2 agonists + antimuscarinic agents + inhaled Corticosteroids	1 (0.6%)	0 (0%)	1 (100.0%)	0.317
Inhaled Corticosteroids	33 (18.8%)	9 (5.1%)	24 (72.7%)	< 0.001
Antimuscarinic agents (short acting)	6 (3.4%)	2 (1.1%)	4 (66.7%)	0.153
Antimuscarinic agents (long acting)	31 (17.6%)	22 (12.5%)	9 (29.0%)	0.180
Anti-leukotrienes	47 (26.7%)	27 (15.3%)	20 (42.6%)	0.009
Others	2 (1.1%)	1 (0.6%)	1 (50.0%)	0.562

	Wash-out period	Post-intervention period	Absolute (%) reduction	p-value^†^
Beta-2 agonists				
Short acting	20 (11.4%)	22 (12.5%)	− 2 (− 10.0%)	0.742
Long acting	0 (0%)	1 (0.6%)	− 1 (–)	0.317
Extra-long acting	0 (0%)	0 (0%)		
Beta-2 agonists + inhaled Corticosteroids	118 (67.1%)	103 (58.5%)	15 (12.7%)	0.098
Beta-2 agonists + antimuscarinic agents	1 (0.6%)	2 (1.1%)	− 1 (− 50.0%)	0.562
Beta-2 agonists + antimuscarinic agents + inhaled Corticosteroids	1 (0.6%)	0 (0%)	1 (100.0%)	0.317
Inhaled Corticosteroids	18 (10.2%)	9 (5.1%)	9 (50.0%)	0.072
Antimuscarinic agents (short acting)	1 (0.6%)	2 (1.1%)	− 1 (− 50.0%)	0.562
Antimuscarinic agents (long acting)	21 (11.9%)	22 (12.5%)	− 1 (− 4.8%)	0.871
Anti-leukotrienes	36 (20.5%)	27 (15.3%)	9 (25.0%)	0.211
Others	1 (0.6%)	1 (0.6%)	0 (0.0%)	1.000

^†^P-value for the comparisons of outcome measures between pre-intervention and post-intervention period (i.e., p-value of χ^2^ test for categorical variables)

In regards to OCS use, the number of patients requiring at least one prescription decreased by 64% in the “post-intervention period” compared to the “pre-intervention period” (p < 0.001), Table 1. A significant reduction in OCS claims (48.1%) was also observed between the “post-intervention period” and the wash-out period (p = 0.001), Table 1.

The number of patients requiring at least one exacerbation-related hospital admission (hospitalization or ER access) decreased by 44.4% in the 12 months after Dupilumab initiation compared to the pre-intervention period (p = 0.28), without any change in the number of hospital admissions in patients with at least one admission, Table 1.

When comparing the overall use of anti-asthmatic drugs other than Dupilumab and hospital admissions between the 6 months immediately before and after Dupilumab prescription (wash-out *vs* post-intervention period) no differences were observed, with the exception of a marginal higher number of patients with at least one hospital admission in the wash-out period compared to the following one (8.0% *vs* 2.9%, p = 0.04), Table 1.

The number of patients starting Dupilumab stratified by month of initiation are summarized in Additional file 1: Figure S3. The great majority of patients in our cohort (146, 83.0%) started the medication between March and June 2021.

Treatment with Dupilumab was considered discontinued if patients had not received any drug claim over a continuous period of 60 days: in our cohort 14 out of 176 patients (8%) discontinued the pharmacological therapy at a mean (SD) of 82.7 (32.9) days.

The overall healthcare costs had a tenfold increase between the pre-intervention and post-intervention period (from 699.50 to 6783.50 Euros), Table 3. The overall increase in expenditures was mainly led by the cost of Dupilumab, with a slight increase also in the costs of the outpatient services, while the overall costs for anti-asthmatic drugs other the biologics showed a marginal decrease. In regards to the expenditures connected to hospitalizations and ER accesses, we observed a reduction although not statistically significant.

**Table 3:** Mean (SD) NHS costs in Euros per patient in the pre-intervention and post-intervention period

	Pre-intervention period	Post-intervention period	p-value^†^
Hospitalizations	124.8 (744.4)	38.4 (288.8)	0.153
Respiratory	87.9 (664.1)	10.4 (137.6)	0.131
Non respiratory	36.9 (345.9)	28.0 (255.0)	0.783
Emergency room visits	24.2 (142.4)	30.0 (205.9)	0.758
Respiratory	7.3 (62.9)	3.8 (40.0)	0.530
Non respiratory	16.9 (93.3)	26.3 (168.4)	0.519
Drugs	337.7 (427.0)	6394.7 (1673.8)	< 0.001
Dupilumab	0 (0)	6008.2 (1185.7)	< 0.001
Mepolizumab and Benralizumab	0 (0)	0 (0)	–
Specifics*	199.1 (181.5)	160.0 (182.6)	0.045
Others	138.7 (352.5)	226.4 (1263.1)	0.376
Outpatient services	212.8 (370.9)	320.4 (401.5)	0.009
Respiratory	13.1 (36.9)	35.6 (79.0)	0.001
Non respiratory	199.7 (360.6)	284.9 (384.6)	0.033
Total	699.5 (1189.4)	6783.5 (1858.0)	< 0.001

*Anti-asthmatic drugs other than biologics. ^†^P-value for the comparisons of outcome measures between pre-intervention and post-intervention period (i.e., p-value of χ^2^ test for categorical variable or of the Student’s t-test for the means of paired samples for continuous variables). ^§^Hospital admissions, also including ER accesses, for asthma exacerbations. ^¥^On patients who experienced at least one hospital admission, or ER access, for asthma exacerbations

Finally, a cohort of 176 comparators was identified. Differences in the use of anti-asthmatic drugs other than biologics including OCS and the occurrence of hospital admissions between the corresponding 6 months in the prior year (pre-intervention period) and the matching dates of the post-intervention period were tested and reported in Additional file 2: Table S2. Similarly to the main cohort’s results, we observed a significant reduction both in the number of patients with at least one prescription of anti-asthmatic drugs and in those with at least one prescription of OCS, although differences were greater in Dupilumab patients (23.6% vs 17.1% for anti-asthmatics and 64.2% vs 54.5% for OCS). Also, similarly to the main cohort, no-evidence of outcome differences emerged among the comparators when analyzing the wash-out *vs* the post-intervention period, Additional file 2: Table S2.

## Discussion

According to the data available from 176 patients with asthma in the large HCU databases from Lombardy, a region of Northern Italy, the initiation of Dupilumab, decreased by 64% the number of patients requiring OCS for asthma control and by 44.4% those requiring exacerbation-related hospital admissions, although the latter without statistical significance, compared to the corresponding period in the pre-dupilumab year.

Similar results were also reported by other real-life cohort studies. *Dupin *et al*.* reported a significant reduction in both daily prednisone dose and annual exacerbations rates in the year following Dupilumab introduction [4]. *Pelaia *et al*.* also found a significant decrease in corticosteroid intake already after 4 weeks from Dupilumab initiation [5]. Nevertheless, the small number of hospital admissions in our cohort (a maximum of 14 in the wash-out period and a minimum of 5 in the post-intervention period) did not allow us to draw definitive conclusions on this outcome.

In our cohort, we also observed other markers of improved asthma control after the initiation of Dupilumab: a significant reduction in the number of patients requiring reliever medications for asthma flare-ups (SABA) and those requiring add-on leukotriene receptor antagonists therapy. However, better disease control may lead patients with asthma to worsen the adherence to maintenance therapy with ICS and/or ICS/LABA, as previously reported by multiple studies [10, 11]. Our results seems to confirm these findings, in fact the percentage of patients with no claims of ICS and ICS/LABA significantly increased in the post-intervention compared to the pre-intervention period (from 19.9 to 41.5% for ICS/LABA association and from 81.1 to 94.9% for ICS alone).

Nevertheless, when comparing the wash-out period (the 6 months immediately preceding the start of Dupilumab) to the post-intervention period, the differences in anti-asthmatic drugs use, including OCS, are no longer observed. The reasons for this discrepancy may be multiple. First of all, a “COVID-19 effect”: the 6-month wash-out period fell for the majority of patients in our cohort during Winter 2021. Autumn 2020 and Winter 2021 overlapped for Northern Italy with the second pandemic wave and the related lock-down. Prior literature showed that social distancing measures favored a reduction in asthma exacerbations and, consequentially, a reduction in the use of medications for asthma flare-ups [12]. Furthermore, the adherence to asthma controller medications during the COVID-19 pandemic has been the subject of conflicting observations, with some studies reporting an improvement in ICS compliance and a reduction in salbutamol use during the pandemic, [13] and others describing a reduced adherence to both asthma controller and reliever medications [14]. Therefore, we speculate that the COVID-19 pandemic may have impacted on the use of anti-asthmatic medications and exacerbations rate in our cohort, particularly in the “wash-out” period.

Secondly, although Dupilumab was available for asthma through the Italian NHS since December 2020, it is possible that a small, but not irrelevant, number of patients received the biologic drug in the months/weeks immediately before this date, in consideration of the pharmaceutical industry’s early access programs (not tracked by the RHS databases), and this may have impacted on the reduction of anti-asthmatic drugs claim in the “wash-out” period. Thirdly, a seasonality effect, with the “wash-out period” falling mainly in Winter and the “post-intervention period” falling mainly in Spring–Summer, may have had a role.

Furthermore, we observed in the cohort of asthmatic patients used as comparators a statistical significant reduction of anti-asthmatic drugs between 2020 and 2021, although smaller than in those with Dupilumab. In particular, the reduction in anti-asthmatic drugs use between the 6 months after Dupilumab initiation and the corresponding period in the pre-dupilumab year was 24%, while the reduction for the asthmatic comparators without Dupilumab in the same period was 17%. The factors associated with this reduction, again a possible “COVID-19 effect” or climatic conditions, also acted as possible confounding factors, enhancing the reduction in anti-asthmatic drugs use between pre-intervention and post-intervention period.

Given the recent introduction of Dupilumab in clinical practice for the treatment of severe asthma, tolerability and healthcare costs sustainability remain open questions.

Recent literature suggested a discontinuation rate, either due to patient’s decision, lack of efficacy or adverse events, ranging from 4.7 to 13.4% [4, 15]. We observed a discontinuation rate of 8%, in line with other biological drugs for severe asthma recently evaluated in a cohort of patients with similar characteristics (3% for mepolizumab and 9% for benralizumab) [9].

In our study, we observed a tenfold increase in overall expenditures mainly led by the cost of Dupilumab, and, secondly, by the cost of outpatient services, probably due to the outpatient clinic accesses and procedures for Dupilumab administration. However, despite a reduction in the costs for anti-asthmatic drugs other than biologics, the expenditures connected to hospitalizations and ER accesses did not significantly change between the pre-intervention and post-intervention period, raising concerns on the healthcare sustainability in relation to biologic therapies. Measures to optimize the healthcare costs may include a better selection of both candidates and responders to biologic drugs [16].

Our investigation, despite being based on HCU databases that provide highly accurate data in a very large and unselected population, also has some limitations beyond those inherent the observational studies. A main limitation is that, because of privacy regulations, hospital records were not available for scrutiny, which means that the diagnostic validity of asthma, as well as other specific variables such as dosages of anti-asthmatic drugs, particularly OCS, and pulmonary function tests could not be checked; thus, evaluation of asthma severity was not possible and this may also have impacted on the evaluation of healthcare costs. Another limitation of our study is that data on main comorbid conditions that influenced both the use of Dupilumab and the severity of asthma itself, such as atopic dermatitis and chronic sinusitis with nasal poliposis, were not available. Third, in case of treatment discontinuation, the specific cause, either inefficacy or adverse events, was not available. Fourth, having the study being conducted during the COVID-19 pandemic, we took into account the “COVID-19 effect” as a possible confounder. Finally, since clinical data, including information on asthma severity, such as questionnaires, physical characteristics and lifestyle information were not available in our database and could vary within the study period, we cannot rule out the possibility of these being unmeasured confounders.

These limitations notwithstanding, our findings suggest that Dupilumab reduces OCS, reliever medications (SABA) and leukotriene receptor antagonists use in patients with asthma. Dupilumab discontinuation rate was low (8%), however nonadherence to inhaled maintenance therapy (ICS/LABA and ICS alone) was not irrelevant.

Overall healthcare costs had a tenfold increase between the corresponding 6 months in the prior year and the 6 months after Dupilumab initiation, which was mainly led by the biologic drug cost. Future research studies should be able to involve greater numbers of patients and observe patients for longer follow-ups to assess the long-term impact of Dupilumab, as well as to evaluate cost-effectiveness and sustainability, giving also the possibility to be conducted out of the pandemic period and avoiding other possible confounders.

### Supplementary Information

**Additional file 1: Figure S1.** Asthmatic patients starting Dupilumab stratified by month of initiation.

**Additional file 2: Table S1.** Diagnostic and therapeutic (ICD-9-CM and ATC) codes used in the current study for drawing records and fields from Healthcare Utilization databases. Lombardy, Italy, 2020–2021. **Table S2.** Use of specific healthcare services during the 6 months after the matching date (follow-up period), during the corresponding 6 months of the prior year (baseline period) and during the 6 months preceding the matching date (wash-out period) among subjects who did not use Dupilumab and were 1:1 matched to those who used it.

## Data Availability

The data that support the findings of this study are available from Lombardy Region, but restrictions apply to the availability of these data, which were used under license for the current study, and so are not publicly available. Data are however available from the Lombardy Region upon reasonable request.

## Abbreviations

ER: Emergency-room
HCU: Healthcare utilization
ICS: Inhaled corticosteroids
LABA: Long-acting β2-agonists
MCS: Multisource Comorbidity Score
NHS: National Health Service
OCS: Oral corticosteroids
RCT: Randomized controlled trials
RHS: Regional Health Service
SABA: Short-acting β2-agonists
SD: Standard deviation
