# Supplementary figures and images for: Effectiveness and economic impact of Dupilumab in asthma: a population-based cohort study
Source: Respir Res. 2023 Mar 7;24:70. doi: 10.1186/s12931-023-02372-y (PMC9990964; doi:10.1186/s12931-023-02372-y)

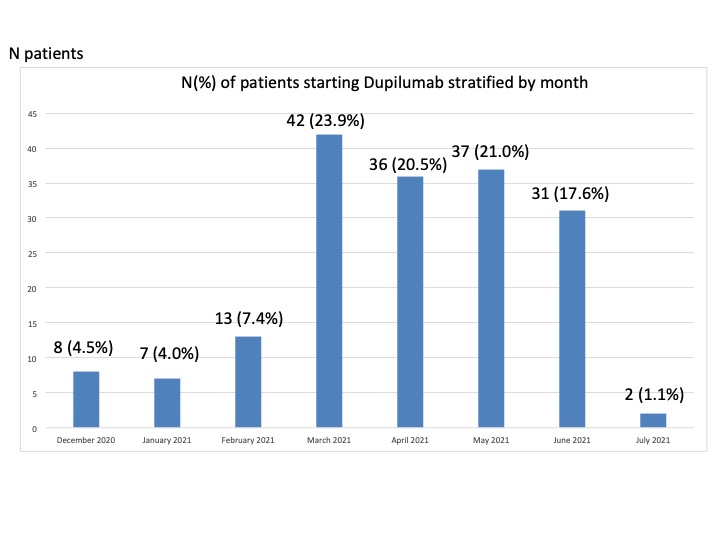

Supplement: Supplementary file 1 — Additional file 1: Figure S1. Asthmatic patients starting Dupilumab stratified by month of initiation. [file 12931_2023_2372_MOESM1_ESM.jpg]
