# Supplementary material for: Effectiveness and economic impact of Dupilumab in asthma: a population-based cohort study
Source: Respir Res. 2023 Mar 7;24:70. doi: 10.1186/s12931-023-02372-y (PMC9990964; doi:10.1186/s12931-023-02372-y)
Supplement: Supplementary file 2 — Additional file 2: Table S1. Diagnostic and therapeutic (ICD-9-CM and ATC) codes used in the current study for drawing records and fields from Healthcare Utilization databases. Lombardy, Italy, 2020–2021. Table S2. Use of specific healthcare services during the 6 months after the matching date (follow-up period), during the corresponding 6 months of the prior year (baseline period) and during the 6 months preceding the matching date (wash-out period) among subjects who did not use Dupilumab and were 1:1 matched to those who used it. [file 12931_2023_2372_MOESM2_ESM.docx]

**Supplementary Table S1.** Diagnostic and therapeutic (ICD-9-CM and ATC) codes used in the current study for drawing records and fields from Healthcare Utilization databases. Lombardy, Italy, 2020-2021.

| **DISEASES** | |
| --- | --- |
|  | **ICD-9-CM code** |
| Asthma | 493.*, J45.* |
| **Exclusion criteria** |  |
| Pneumonia | 480.* – 488.* |
| Chronic pulmonary diseases | 518.89, 496.* |
| Chronic obstructive pulmonary disease (COPD) | 491.*, 492.*, 496.* |
| Bronchiectasis | 494.* |
| Diffuse infiltrative pulmonary disease | 508.1, 515, 516.3, 518.89, 714.81, 770.7 |
| Cystic fibrosis | 277.0 |
| Obstructive sleep apnoea syndrome | 327.23 |
| Pulmonary arterial hypertension | 416.0 |
| Lung cancer | 162.*, 197.0, 231.2, 212.3, 235.7, 239.1 |
| Eosinophilic Granulomatosis with Polyangiitis (EGPA) | 447.6 |
| **Outcomes** |  |
| Hospital admissions for asthma exacerbations | 460 - 519 |
| **Covariates/Baseline characteristics** |  |
| Cancer | 140, 208 |
| Paget disease | 731 |
| Cushing syndrome | 255 |
| Hyperthyroidism / Hypothyroidsm | 242, 252 |
| Celiac disease | 579 |
| Parkinson disease | 3220 |
| Orthostatic hypotension | 4580 |
| Blindness | 369 |
| Dementia | 290, 294, 331, 334, 335 |
| Cerebrovascular diseases | 430 – 438 |
| Chronic liver failure | 571 |
| Chronic renal failure | 585, v56 |
| **DRUGS** | |
|  | **ATC codes** |
| **Biological drug** |  |
| Dupilumab | D11AH05 |
| **Exclusion criteria** |  |
| Mepolizumab | R03DX09 |
| Reslizumab | R03DX08 |
| Benralizumab | R03DX10 |
| Omalizumab | R03DX05 |
| **Outcomes** |  |
| Beta-2 agonists |  |
| Short acting | R03AC02, R03AC03 |
| Long acting | R03AC13, |
| Extra-long acting | R03AC18, R03AC19 |
| Extra | R03AK01, R03AK04 |
| *…* + inhaled Corticosteroids | R03AK06, R03AK07, R03AK08, R03AK09, R03AK10, R03AK11, R03AK12, R03AK13 |
| *…* + antimuscarinic agents | R03AL02, R03AL03, R03AL05, R03AL06 |
| *…* + antimuscarinic agents + inhaled Corticosteroids | R03AL09 |
| Inhaled Corticosteroids | R03BA01, R03BA02, R03BA03, R03BA04, R03BA05, R03BA06, R03BA07, R03BA08, R03BA09 |
| Antimuscarinic agents (short acting) | R03BB01, R03BB02, R03BB03 |
| Antimuscarinic agents (long acting) | R03BB04, R03BB05, R03BB06, R03BB07, R03BB54 |
| Anti-leukotrienes | R03DC01, R03DC03 |
| Others | R03DX07, R03DA04, R03BC01, R03BC03 |
| **Systemic glucorticoids** | QH02AB30, QH02AB56, QH02AB57, QH02AB90, H02BX01, QH02BX90, H02AB, QH02AB30, QH02AB56,  QH02AB57, QH02AB90, H02B, QH02BX90 |
| **Covariates/Baseline characteristics** |  |
| Antidiabetics | A10 |
| Antithrombotics | B01 |
| Statins | C01AA |
| Antiarrrhythmics | C01B |
| Nitrats | C01DA |
| Antihypertensives | C02, C03, C07, C08, C09 |
| Antiepileptics | N03 |
| Antidepressants | N06A |
| Antineoplastics | H03B |
| **CO-PAYMENTS EXEMPTIONS** | |
|  | **Exemption codes** |
| Asthma | 007.* |
|  |  |

**Supplementary Table S2.** Use of specific healthcare services during the 6 months after the matching date (follow-up period), during the corresponding 6 months of the prior year (baseline period) and during the 6 months preceding the matching date (wash-out period) among subjects who did not use Dupilumab and were 1:1 matched to those who used it.

|  | **Pre-intervention**  **period** | **Post-intervention**  **period** | **Absolute (%) reduction** | **p-value†** |
| --- | --- | --- | --- | --- |
|  |  |  |  |  |
| Patients with at least one asthma prescription | 123 (69.9%) | 102 (58.0%) | 21 (17.1%) | 0.02 |
| Mean number of asthma prescriptions per patient (*patients with at least one Rx*) | 10.8 (9.6) | 5.7 (4.7) | 5.1 (47.2%) | <0.001 |
|  |  |  |  |  |
| Patients with at least one systemic glucorticoid prescription | 44 (25.0%) | 20 (11.4%) | 24 (54.5%) | <0.01 |
| Mean number of glucorticoid prescriptions per patient (*patients with at least one Rx*) | 2.6 (3.6) | 2.0 (1.7) | 0.6 (23.1%) | 0.71 |
|  |  |  |  |  |
| Patients with at least one hospital admissions**^§^** for asthma exacerbations | 8 (4.6%) | 10 (5.7%) | 2 (25.0%) | 0.63 |
| Mean number of hospital admissions**^§^** for asthma exacerbations**^¥^** | 1.0 (0.0) | 1.7 (1.6) | 0.7 (0.7%) | 0.21 |
|  | **Wash-out**  **period** | **Post-intervention**  **Period** | **Absolute (%) reduction** | **p-value†** |
|  |  |  |  |  |
| Patients with at least one asthma prescription | 99 (56.3%) | 102 (58.0%) | 3 (3.0%) | 0.75 |
| Mean number of asthma prescriptions per patient (patients with at least one Rx) | 4.6 (4.3) | 5.7 (4.7) | 1.1 (23.9%) | 0.07 |
|  |  |  |  |  |
| Patients with at least one systemic glucorticoid prescription | 25 (14.2%) | 20 (11.4%) | 5 (19.7%) | 0.42 |
| Mean number of glucorticoid prescriptions per patient (patients with at least one Rx) | 2.0 (1.6) | 2.0 (1.7) | 0.0 (0.0%) | 1.00 |
|  |  |  |  |  |
| Patients with at least one hospital admissions§ for asthma exacerbations | 5 (2.8) | 10 (5.7%) | 5 (50%) | 0.19 |
| Mean number of hospital admissions§ for asthma exacerbations¥ | 1.2 (0.4) | 1.7 (1.6) | 0.5 (41.7%) | 0.39 |

**†** P-value for the comparisons of outcome measures between baseline and follow-up period (i.e., p-value of χ2 test for categorical variable or of the Student’s t-test for the means of paired samples for continuous variables). **§** Hospital admissions, also including ER accesses, for asthma exacerbations. **¥** On patients who experienced at least one hospital admission, or ER access, for asthma exacerbations.
